# Translation and validation of the Amsterdam preoperative anxiety and information scale (APAIS) in Serbia

**DOI:** 10.1002/brb3.2462

**Published:** 2021-12-15

**Authors:** Ksenija Jovanovic, Nevena Kalezic, Sandra Sipetic Grujicic, Vladan Zivaljevic, Milan Jovanovic, Milica Savic, Zoran Bukumiric, Marko Dragas, Milos Sladojevic, Ranko Trailovic, Igor Koncar, Lazar Davidovic

**Affiliations:** ^1^ Center for Anesthesiology and Resuscitation University Clinical Center of Serbia Belgrade Serbia; ^2^ Faculty of Medicine University of Belgrade Belgrade Serbia; ^3^ Institute of Epidemiology Faculty of Medicine, University of Belgrade Belgrade Serbia; ^4^ Center for Endocrine Surgery University Clinical Center of Serbia Belgrade Serbia; ^5^ Institute of Medical Statistics and Informatics Faculty of Medicine, University of Belgrade Belgrade Serbia; ^6^ Clinic for Vascular and Endovascular Surgery University Clinical Center of Serbia Belgrade Serbia

**Keywords:** Amsterdam preoperative anxiety and information scale (APAIS), preoperative anxiety, reliability, validity, visual analogue scale for anxiety (VAS‐A)

## Abstract

**Objectives:**

Preoperative anxiety is common and might affect surgical treatment outcomes. The aim was to translate and validate the Serbian version of the Amsterdam Preoperative Anxiety and Information Scale (APAIS).

**Methods:**

Following translation and initial evaluation, the Serbian version (S‐APAIS) was administered to 385 patients. Internal consistency, construct validity, prognostic criteria validity, and concurrent validity between S‐APAIS and Visual Analogue Scale for Anxiety (VAS‐A) were evaluated.

**Results:**

Factor analysis revealed two factors: APAIS‐anesthesia (items 1, 2, 3) and APAIS‐procedure (items 4, 5, 6). The whole scale, APAIS‐anesthesia, and APAIS‐procedure subscales showed an adequate level of internal consistency (Cronbach's *α*s: 0.787, 0.806, and 0.805, respectively). High concurrent validity was observed between APAIS‐anesthesia and VAS‐A (ρ = 0.628, *p* < .001). A moderate correlation was found between APAIS‐procedure and VAS‐A scale (ρ = 0.537, *p* < .001). At the cut‐off point of 9, the area under the curve (AUC) of APAIS‐anesthesia was 0.815 (95% CI: 0.77–0.85, *p* < .001). For the APAIS‐procedure, AUC was 0.772 (95% CI: 0.73–0.81, *p* < .001) at the cut‐off point of 8.

**Conclusion:**

The structure of S‐APAIS substantially differs from the original and allows separate measurement of anesthesia‐ and procedure‐related anxieties. S‐APAIS is a comprehensive, valid, and reliable instrument for the measurement of preoperative anxiety.

## Graphical abstract

Preoperative anxiety represents an important issue and should be routinely assessed. The APAIS scale is a valid tool for the assessment of preoperative anxiety. Serbian APAIS version has good psychometric properties and modified structure and can distinguish between surgery‐ and anesthesia‐related anxiety.

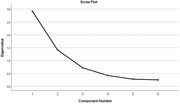

## 1 INTRODUCTION

Anxiety can be defined as an aversive feeling which arises from the anticipation of a potentially unfavorable, risky, or unpleasant event or outcome. It is characterized by negative emotional reactions and intense physical manifestations (Hyde et al., 2019). The concept of preoperative anxiety refers to an unpleasant feeling of worry in a patient undergoing surgical treatment. It is usually related to the perception of the forthcoming operation or anesthesia, pain, hospitalization, and disease itself. It is very common and can be seen in up to 94% of patients prior surgery (Aust et al., 2018). Besides the fact that preoperative anxiety is marked as by far the worst aspect of treatment by surgical patients (Walker et al., 2016), it can significantly affect surgical treatment outcomes (Williams et al., 2013). This justifies the need for routine preoperative assessment of anxiety levels.

Several tools are available for the identification of patients with preoperative anxiety. The Amsterdam Preoperative Anxiety and Information Scale (APAIS) was developed in 1996 by Moerman et al. (1996) and the original Dutch version has been translated and validated into many reliable versions in several languages (Maurice‐Szamburski et al., 2013; Mohd et al., 2015; Vergara‐Romero et al., 2017; Wu et al., 2020). Nowadays, APAIS has gained widespread use, since contemporary literature offers strong evidence of its validity and reliability (Aust et al., 2018). Some authors even consider it as a “gold standard” for preoperative anxiety measurement (Eberhart et al., 2020). To date, the Serbian version does not exist. Therefore, the aim of the present study was to perform translation of the APAIS and to evaluate its validity and reliability for the Serbian population.

## 2 METHODS

The study was conducted during the period from February 1 to October 1, 2019. Consecutive patients who were scheduled for elective vascular surgical procedures (aortic, carotid, and surgery of peripheral arteries) during the eight‐month period were enrolled in the study. Procedures were performed under both general and regional anesthesia. The following patients were excluded from the study: patients under the age of 18, those who have already undergone some kind of vascular surgery, patients with an anxiety disorder diagnosed preoperatively, those with whom it was not possible to establish meaningful communication, as well as those who refused voluntarily to participate in the study. Written consent was obtained from all the study participants and the study was approved by the Ethics Committee of the Faculty of Medicine, University of Belgrade, Serbia (No 1550/V‐18).

Patients were asked to fill out the questionnaires one day prior to the surgery, under the supervision of one of six doctors from the anesthesia unit (three specialists and three anesthesia residents). Besides two specific anxiety questionnaires (APAIS and Visual Analogue Scale for Anxiety – VAS‐A), patients were asked to complete the general questionnaire as well, with questions regarding basic demographic characteristics of patients: gender, age, marital status, educational background, employment status, number of children and household members, and socioeconomic conditions.

APAIS is a self‐reported questionnaire, which can quickly assess the level of preoperative anxiety. The scale contains six questions, grouped into two components: anxiety subscale, which measures anxiety related to anesthesia and surgery (questions 1, 2, 4, and 5) and the need for information subscale (questions 3 and 6), assessing the desire for information regarding anesthesia and surgery. Questions are scored based on Likert's method from 1 (“not at all”) to 5 (“extremely”). For the subscale related to the anxiety, the total score ranges from 4 to 20, while for the part of the scale related to the need for information the range is from 2 to 10. A higher score speaks in favor of a higher level of anxiety and a greater need for information. According to the part of the scale related to preoperative anxiety, a patient with a score ≥11 experiences anxiety; according to the part of the scale related to the need for information related to anesthesia and surgery, patients are classified into those who have little or no need for information (score 2–4), those who have an average (score 5–7) and those who have high information requirements (score 8–10) (Moerman et al., 1996).

VAS‐A consists of a 100 mm long horizontal line, which is marked with 0 (“not anxious at all”) at its left and with 100 (“extremely anxious”) at its right end (Abend et al., 2014). The patients were asked to draw a vertical line and thus mark the level of their anxiety at the time of the interview. A line drawn closer to the right end indicates a greater degree of anxiety. Although the cut‐off point is not precisely established (Stamenkovic et al., 2018), a VAS‐A value > 70 mm correlates with a high level of anxiety (Hernandez et al., 2015).

Permission to translate the APAIS and to validate the Serbian version was obtained from the author of the original scale (N. Moerman). The translation and adaptation were performed according to the internationally accepted methodology for translation and cross‐cultural adaptation of questionnaires (Sousa & Rojjanasrirat, 2011) and included several steps. First, a “forward translation” (from English to Serbian) was performed by two independent professional translators. Those two initial translations were merged into one by the members of the expert team (the lead investigator, an epidemiologist, two professional translators, and one clinician). This version was as close as possible to the original APAIS regarding its semantic and conceptual characteristics but at the same time the most appropriate for the Serbian cultural environment. This version was used for the “backward translation,” from Serbian to English. Then, the expert team discussed this version and compared it with the original APAIS. The author of the original scale approved the APAIS backward translation. Since differences that could change the meaning of the questions were not observed, no further changes have been made and a prefinal Serbian version was obtained. In order to perform an initial evaluation and to assess understanding of the questionnaire in the Serbian population, a small sample pilot study was conducted. Thirty patients were asked to fill‐out a simple questionnaire which consisted of five questions regarding clarity, unpleasantness, durability, extensiveness, and importance of the subject of the Serbian version of APAIS. Questions were graded on a Likert scale from 1 (“not at all”) to 5 (“extremely”). The results were reviewed among the expert team members. No further corrections were needed, so this stage finally led to the production of the definitive Serbian version of the APAIS scale (S‐APAIS).

Demographic characteristics of patients were analyzed using descriptive statistics methods. Continuous variables are presented as means ± standard deviation, while categorical variables are presented as absolute numbers with percentages. The Kolmogorov–Smirnov test was used to check the normality of the distribution of continuous variables.

The psychometric properties of the Serbian version of the APAIS scale included analysis of the following domains: internal consistency, construct validity, concurrent validity, and prognostic criteria validity. Internal consistency was assessed using Cronbach's α coefficient. The scale was considered reliable if Cronbach's α coefficient was > 0.7. To examine whether the original APAIS scale maintains its factor structure in the data gathered among the Serbian population, a confirmatory factor analysis (CFA) was performed. An acceptable model fit was based on the following thresholds of fit indices: chi‐square‐degrees of freedom ratio (*χ* ^2^/DF) lower than 2, the root mean square error of approximation (RMSEA) lower than 0.08, the goodness fit index (GFI) higher than 0.9, the adjusted GFI (AGFI) higher than 0.9, normed fit index (NFI) higher than 0.9, and comparative fit index (CFI) higher than 0.9 (Hu & Bentler, 1999). Subsequently, to determine a viable factor structure, an exploratory factor analysis (EFA) was conducted, using the principal component analysis and Varimax rotation method. The appropriateness of data for EFA was assessed using Kaiser–Meyer–Olkin's measure of sampling adequacy and Bartlett's test of sphericity. Concurrent validity between S‐APAIS and VAS‐A scale was expressed through Spearman's correlation coefficient (ρ). In order to investigate the prognostic criteria validity of S‐APAIS, a receiver operating curve (ROC) analysis was conducted. VAS‐A scale was used as “gold standard” with a score over 70 mm as a cut‐off point for the detection of anxious patients.

Statistical analysis was performed using SPSS 22.0 (Chicago, IL, USA) and the Analysis of Moment Structure (AMOS) Version 21.0.0 (Meadville, PA, USA). A *p* value < .05 was considered significant.

## 3 RESULTS

The final Serbian version of the APAIS is shown in Table 1. During an 8‐month study period, a total of 402 questionnaires were distributed. Seventeen patients were excluded from the study since they did not meet inclusion criteria: five have already undergone some kind of vascular surgical intervention, 11 have had anxiety disorder, and one patient refused to participate in the study. The definitive study sample included 385 patients, with a male to female ratio of 3.8:1. The mean age of patients was 67.1 ± 7.4 years. The majority were married (70.4%), with two children (47.8%) and retired (70.9%). More than one‐sixth of patients lived alone (17.4%) and almost half of the study sample reported fair socioeconomic conditions (46.8%). Basic sociodemographic characteristics of patients are presented in Table 2.

**TABLE 1:** The original Amsterdam Preoperative Anxiety and Information Scale and the Serbian version

**Original APAIS**	**Serbian APAIS**
1. I am worried about the anesthetic.	1. Zabrinut(a) sam zbog anestezije.
2. The anesthetic is on my mind continually.	2. Stalno mislim na anesteziju.
3. I would like to know as much as possible about the anesthetic.	3. Voleo(la) bih da znam što više o anesteziji.
4. I am worried about the procedure.	4. Zabrinut(a) sam zbog operacije.
5. The procedure is on my mind continually.	5. Stalno mislim na operaciju.
6. I would like to know as much as possible about the procedure.	6. Voleo(la) bih da znam što više o operaciji.

**TABLE 2:** Basic sociodemographic and clinical characteristics of patients

Characteristics	Number (Percentage)
**Gender**	
Male	305 (79.2%)
Female	80 (20.8%)
**Education**	
Not literate / Incomplete primary school	15 (3.9%)
Primary	72 (18.7%)
High school	214 (55.6%)
University degree	81 (21.0%)
Master's / PhD	3 (0.8%)
**Marital status**	
Single (never married)	33 (8.6%)
Married/cohabiting	271 (70.4%)
Widowed	52 (13.5%)
Separated/divorced	29 (7.5%)
**Employment status**	
Employed	71 (18.4%)
Unemployed	41 (10.7%)
Retired	273 (70.9%)
**Socioeconomic conditions**	
Good	156 (40.5%)
Fair	180 (46.8%)
Bad	49 (12.7%)
**Children**	
No	54 (14.0%)
1	80 (20.8%)
2	184 (47.8%)
3	52 (13.5%)
>3	15 (3.9%)
**Number of household members**	
Lives alone	67 (17.4%)
Multi‐member household	318 (82.6%)
**Surgery type**	
Aortic	143 (37.1%)
Carotid	157 (40.8%)
Peripheral arteries	85 (22.1%)

The majority of patients (93.3%) accepted S‐APAIS well and rated it as understandable. The same percentage of patients stated that questions were not unpleasant. According to 96.7% of patients, questions were not time‐consuming, while 86.7% of patients perceived the questionnaire as not too extensive. Also, 60% of patients considered that the topic of the questionnaire is important.

**FIGURE 1:**
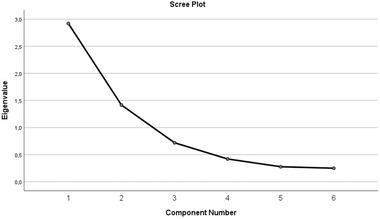
Scree plot

Construct validity of the Serbian version of APAIS was assessed using factor analysis. Results of CFA showed unacceptable values of global fit indices: χ^2^ = 364.34, *p* < .001, χ^2^/DF = 45.54, RMSEA = 0.341, GFI = 0.756, AGFI = 0.360, NFI = 0.613, and CFI = 0.615. Thus, a two‐factor model of the original APAIS scale did not provide a good fit to the data and was rejected. The Kaiser–Meyer–Olkin measure of sampling adequacy score was 0.71 and the results of Bartlett's test of sphericity were statistically significant (χ^2^ = 934.778, *p* < .001), suggesting that data was suitable for EFA. An exploratory factor analysis revealed two factors, with eigenvalue over 1 and which together explained 72.3% of the total variance. The scree plot test also suggested a two factors model. (Figure 1) The first three items had the highest association with the first component, while items 4–6 were associated with the second component. The eigenvalue for the first factor, anxiety and need for information related to anesthesia was 2.92 (APAIS‐anesthesia = items 1, 2, 3) and it explained 48.7% of the variance. The eigenvalue for the second factor, anxiety and need for information related to the procedure was 1.415 (APAIS‐procedure = items 4, 5, 6) and it explained 23.6% of the variance. (Table 3).

**TABLE 3:** Factor structure, exploratory variance values, and eigenvalues of the S‐APAIS

Factors	Items	Cronbach's α	Factor loadings
Factor 1	1	0.806	0.886
	2		0.880
	3		0.738
Factor 2	4	0.805	0.850
	5		0.878
	6		0.771
Total Cronbach's α		0.787	

	Exploratory variance values of factors	Eigenvalues
Factor 1	48.661	2.920
Factor 2	23.584	1.415
Total variance	72.245

Serbian version of the APAIS scale has an adequate level of internal consistency, based on the value of Cronbach's α coefficient of 0.787. Furthermore, both extracted factors showed high level of internal consistency (APAIS‐anesthesia: Cronbach's α = 0.806 and APAIS‐procedure: Cronbach's α = 0.805). (Table 3) Item‐total analysis showed that “Cronbach's α if item deleted” ranged from 0.742 to 0.774 and no correlation value less than 0.30 (Table 4).

**TABLE 4:** Internal consistency and homogeneity of the Serbian version of the Amsterdam Preoperative Anxiety and Information Scale (APAIS)

Items	Scale mean if item deleted	Scale variance if item deleted	Corrected item‐total correlation	Cronbach's α if the item is removed
1	12.51	27.803	0.542	0.754
2	12.77	27.907	0.591	0.743
3	12.65	29.232	0.475	0.770
4	12.38	27.498	0.590	0.742
5	12.38	26.976	0.573	0.746
6	12.75	29.555	0.456	0.774

**FIGURE 2:**
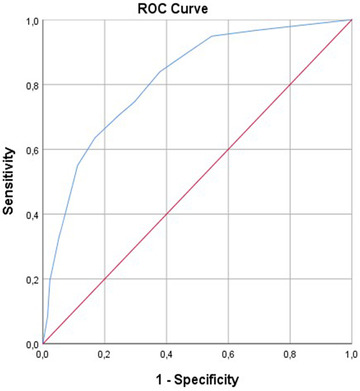
Predictive validity of APAIS‐anesthesia

**FIGURE 3:**
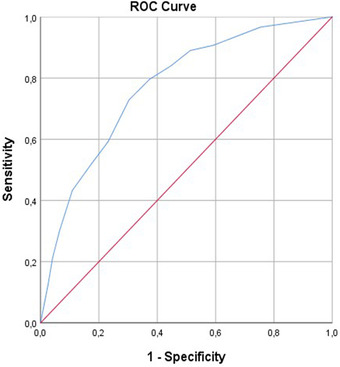
Predictive validity of APAIS‐procedure

Prognostic criteria validity of S‐APAIS subscales was estimated by ROC curve, using the VAS‐A score over 70 mm as a reference point for anxiety. At the cut‐off point of 9, the area under the curve (AUC) of APAIS‐anesthesia was 0.815 (95% CI: 0.77–0.85, *p* < .001), with a sensitivity of 63% and specificity of 83%. (Figure 2) For the APAIS‐procedure subscale, AUC was 0.772 (95% CI: 0.73–0.81, *p* < .001), with a cut‐off point of 8, sensitivity of 72%, and specificity of 69%. (Figure 3)

According to the APAIS‐anesthesia subscale, 31.2% of patients experienced anesthesia‐related anxiety preoperatively (score > 9), and based on the APAIS‐procedure subscale 43.4% of patients were anxious due to forthcoming surgery (score > 8). On the other hand, when assessed through the VAS‐A scale, 30.6% of patients experienced preoperative anxiety—both anesthesia—and surgery‐related (score ≥ 70 mm). High concurrent validity was observed between APAIS‐anesthesia and VAS‐A scale (Spearman's correlation coefficient ρ = 0.628, *p* < .001), while a moderate correlation was found between APAIS‐procedure and VAS‐A scale (Spearman's correlation coefficient ρ = 0.537, *p* < .001).

## 4 DISCUSSION

The main finding of the present study is that the structure of the Serbian version substantially differs from the original APAIS scale. As proposed by Moerman et al. (1996), the original APAIS is characterized by two sub‐dimensions: (1) scale related to anxiety, with items 1, 2, 4, and 5, and (2) scale related to the desire for information, with questions 3 and 6. Original APAIS possesses excellent psychometric characteristics and represents a valid tool for preoperative anxiety evaluation. The main (and perhaps the only) limitation of the original APAIS scale is the fact that it cannot differentiate between anesthesia and surgery‐related anxiety, contrary to the authors’ expectations (Moerman et al., 1996). On the other hand, due to its specific structure Serbian version has overcome this limitation: the Serbian version of APAIS also has two factors, but completely different from those in the original questionnaire. Factor 1 refers to the anesthesia‐related anxiety and the need for information (APAIS‐anesthesia) and factor 2, APAIS‐procedure describes anxiety and need for information related to the procedure. Thus, Serbian version of APAIS is characterized by a modified structure and has two independent anxiety subscales, which can measure surgery‐ and anesthesia‐related anxiety separately. Questions 3 and 6, related to the information desire are included in corresponding subscales (anesthesia/procedure).

There are several possible explanations for the specific S‐APAIS structure. One of them probably lies in some cultural characteristics of the Serbian population. For example, this might be attributable to some cultural aspects regarding anxiety and fear in the Serbian population and ways of coping with those feelings. This is supported by the fact that the Serbian version is not only different from the original, but it also differs from the Malay (Mohd et al., 2015), Chinese (Wu et al., 2020), French (Maurice‐Szamburski et al., 2013), Japanese (Nishimori et al., 2002), and Italian (Buonanno et al., 2017) version as well. To the best of our knowledge, Greek validation study is the only study that showed an identical two‐factor structure of the translated and validated APAIS, with clearly separated anesthesia and procedure‐related subscales (Bakalaki et al., 2017). Besides the fact that one scale might perform differently in different populations (Buonanno et al., 2017), the second possible explanation of the novel S‐APAIS model, also seen in Greek study (Bakalaki et al., 2017), possibly lies in similarities between the two nations (Tokić, 2012), while other nations differ significantly from the Serbian population in terms of cultural, emotional, and religious characteristics. Furthermore, according to the reports of the Statistical Office of the Republic of Serbia, our country still has a high percentage of illiterate (2.67%) and people with incomplete primary education (10.83%) (Statistički kalendar Republike Srbije, 2020). A significant percentage of illiterate and people with insufficient education were also present in our study sample. This might have led to the lack of basic knowledge and information regarding surgical processes and anesthetic techniques and contributed to the modified S‐APAIS structure. Also, unlike surgery, which has always been far more appreciated, anesthesia has always been underestimated and marginalized among the Serbian population, perhaps due to more abstract and unfamiliar perspective. This might explain the strict separation of feelings related to surgery and anesthesia and, consequently, a specific structure of S‐APAIS.

This is the first study that reports the process of translation of the APAIS scale into Serbian and its validation among the Serbian population. Results of the present study indicate that the Serbian version of the APAIS scale possesses good psychometric properties. Namely, S‐APAIS shows an adequate level of internal consistency: Cronbach's α coefficient = 0.787. This value is comparable with those reported by other authors (Mohd et al., 2015; Vergara‐Romero et al., 2017; Zeleníková et al., 2017). In the original APAIS study (Moerman et al., 1996), Cronbach's α coefficient was calculated for the two subscales separately and not for the whole scale and since the S‐APAIS structure differs from the original scale significantly, those values cannot be compared. In addition, two subscales of the present study—APAIS‐anesthesia and APAIS‐procedure, are also characterized by a high level of internal consistency (Cronbach's α = 0.806 and 0.805, respectively), suggesting that those anxieties can be measured separately.

Although different authors consider other scales as “gold standard” for the measurement of preoperative anxiety level (Goebel & Mehdorn, 2018; Lemos et al., 2019; Tulloch & Rubin, 2019; Vergara‐Romero et al., 2017), VAS‐A is as efficient and reliable and can be used for both research and clinical purposes (Hernandez et al., 2015; Homzová & Zeleníková, 2015; Kindler et al., 2000). Results of the present study demonstrate strong and moderate correlations between two S‐APAIS subscales—APAIS‐anesthesia and APAIS‐procedure with VAS‐A (ρ = 0.628, *p* < .001 and ρ = 0.537, *p* < .001, respectively). This association describes the external validity of S‐APAIS, suggesting that it can be effectively used for the assessment of preoperative anxiety.

While data regarding the incidence of preoperative anxiety are inconsistent, a vast majority of authors agree that it is very common. In fact, a recent cross‐sectional study by Aust et al. that included over 3000 subjects showed that only 6% of patients do not feel anxious at all during the perioperative period (Aust et al., 2018). Our results show that preoperative anxiety can be seen in over 40% of patients, which is in concordance with the findings of the other authors (Kuzminskaitė et al., 2019; Nigussie et al., 2014; Woldegerima et al., 2018).

Results of prognostic criteria validity testing show that patients who score more than 9 points on the APAIS‐anesthesia subscale have 63% chance of experiencing anesthesia‐related anxiety. Patients with a score ≤9 will not be anxious about anesthesia in 83% of cases. Patients will experience anxiety related to the procedure if they score more than 8 points on the APAIS‐procedure subscale with a probability of 72% and if this score is ≤8, the chance of them not presenting surgery‐related anxiety is 69%. These results are approximate, but not identical to the ones in the study by Bakalaki et al. (2017), who reported the same two‐factor structure of the modified APAIS scale. Specifically, cut‐off points in that study were 6.5 and 8.5 for the APAIS‐anesthesia and APAIS‐procedure subscales, respectively. These differences might partially be explained by smaller sample size, different gender structure (fewer men), younger subjects, and the fact that patients underwent various surgical procedures in the Greek study. The present study included solely vascular surgical patients, so the percentage of “major surgeries”—defined as more severe ones by Bakalaki et al. (2017), is considerably higher, which might also contribute to differences in cut‐off values.

Finally, besides the fact that S‐APAIS is characterized by good psychometric properties, results of the initial evaluation pilot study show that this questionnaire is brief, understandable, and practical. Since there was no significant misinterpretation, lack of understanding, or need for further explanation of the questions, this finding also supports the application of S‐APAIS in preoperative anxiety assessment in the Serbian population.

Limitations of the present study are reflected in some characteristics of the patients. The sample is not homogeneous in terms of gender structure: there were almost four times fewer females than men. This could have led to lower anxiety incidence since the female gender is known as a risk factor for preoperative anxiety (Gonçalves et al., 2016; Laufenberg‐Feldmann & Kappis, 2013). Also, patients in our study were mostly older, unequally distributed according to surgery type, and operated on in a single center. The effects of surgery type, anesthesia techniques, previous anesthetic/surgery experiences, and other clinical characteristics of patients on anxiety level were not taken into account and will be of interest in our future research. The fact that we did not use the State‐Trait Anxiety Inventory (STAI) to assess concurrent validity as most of the previous studies did, may also be considered a weakness of the present study. Still, a higher cut‐off point on the VAS‐A scale (> 70 mm) for the detection of anxiety cases should compensate for this limitation.

Based on the results of the present study, the Serbian version of APAIS is a valid, reliable, easy to administer, and well‐accepted questionnaire that can effectively be used in the assessment of preoperative anxiety. The structure of the Serbian version substantially differs from the original and has two subscales that can clearly distinguish between anxiety related to anesthesia and anxiety related to surgery. In that manner, the practical value of the S‐APAIS scale can be seen: anesthesia‐ and procedure‐related anxieties can be observed and measured separately and selected patients might be addressed individually by the surgeon or anesthetist, thus providing a higher level of care for the patient and lowering the incidence of preoperative anxiety.

## CONFLICT OF INTEREST

The authors declare that they have no conflict of interest.

### PEER REVIEW

The peer review history for this article is available at https://publons.com/publon/10.1002/brb3.2462

## FUNDING INFORMATION

The authors received no funding for this work.

## Data Availability

The data that support the findings of this study are available from the corresponding author upon reasonable request.
